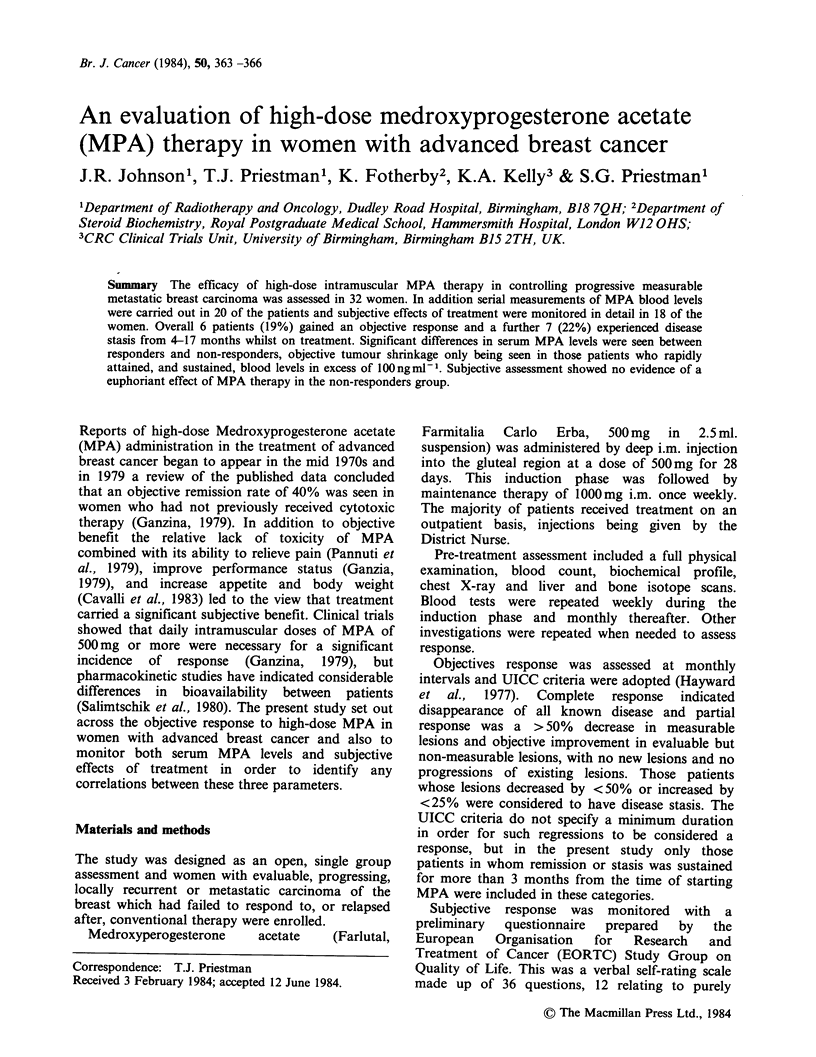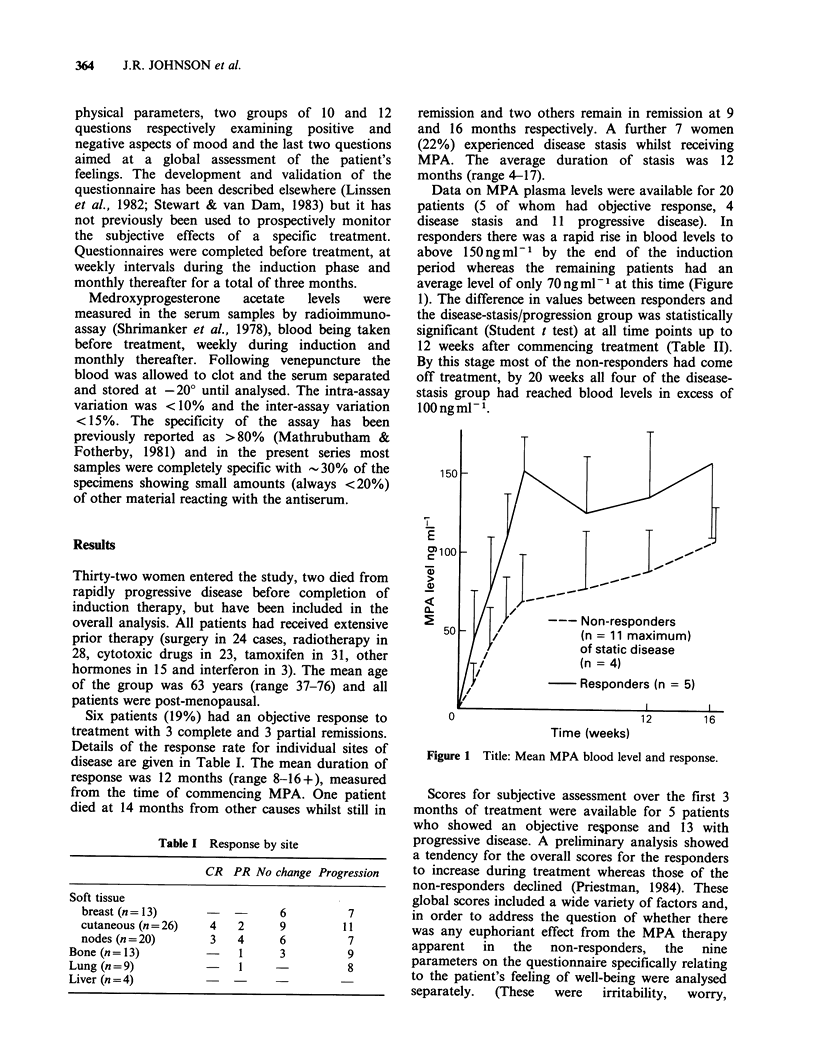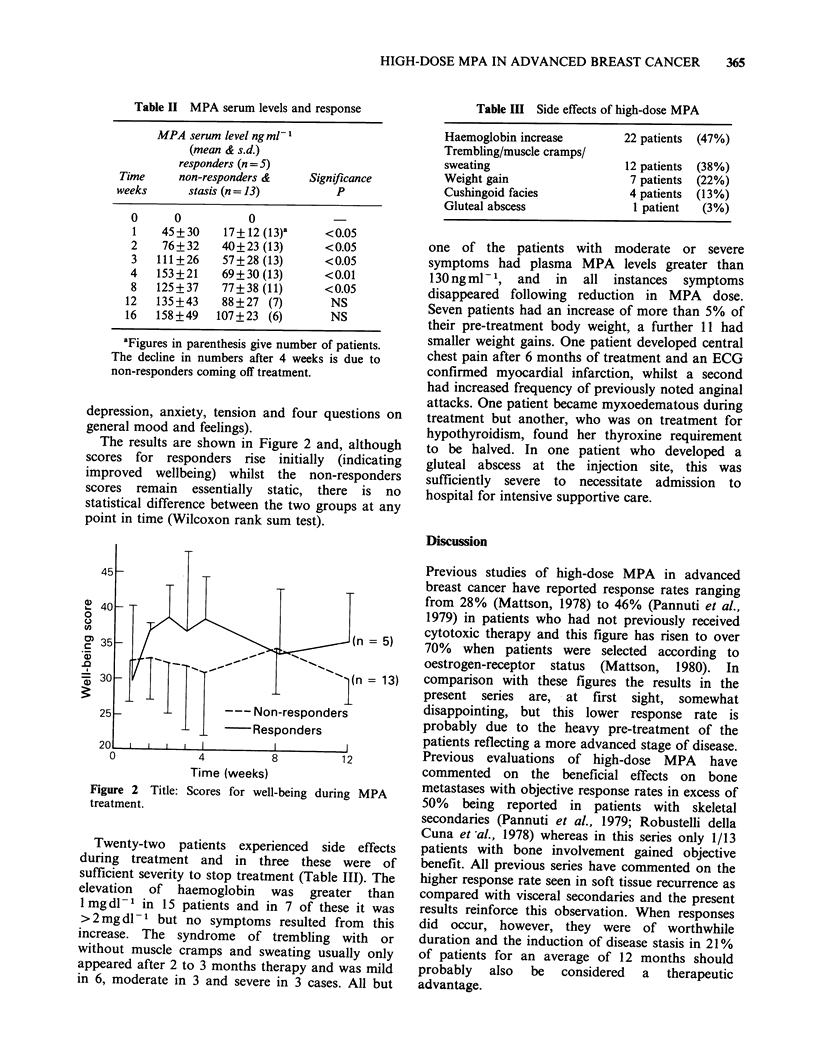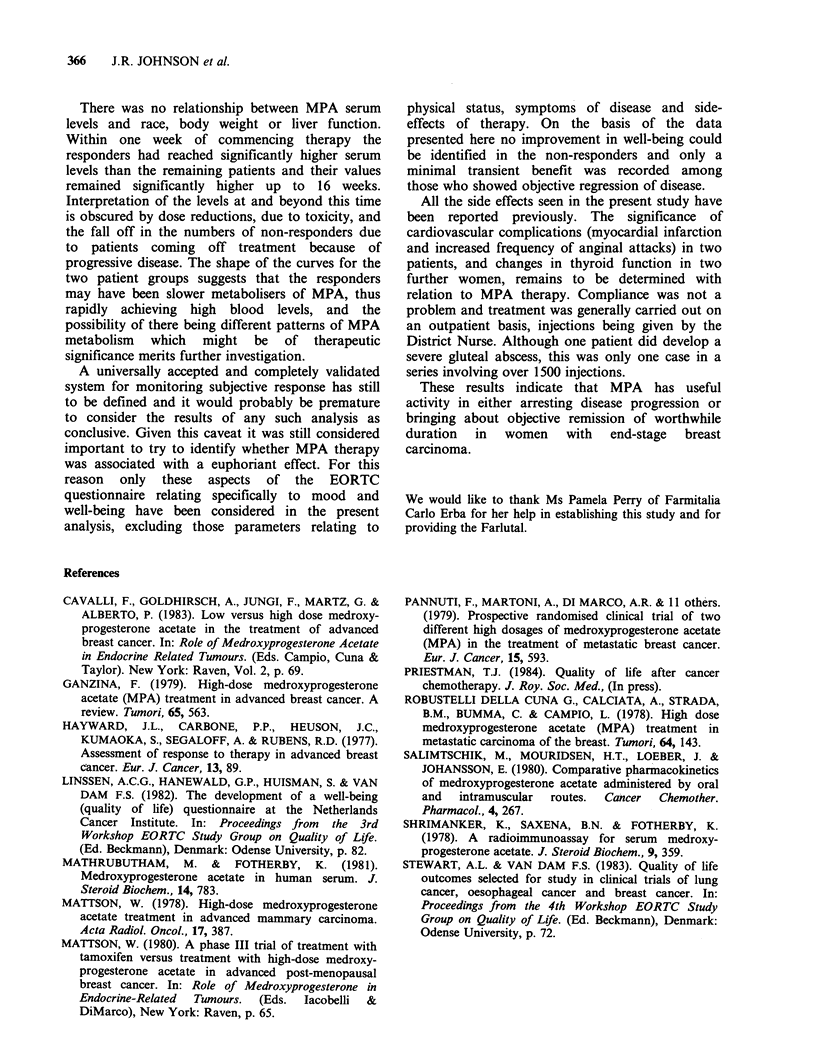# An evaluation of high-dose medroxyprogesterone acetate (MPA) therapy in women with advanced breast cancer.

**DOI:** 10.1038/bjc.1984.184

**Published:** 1984-09

**Authors:** J. R. Johnson, T. J. Priestman, K. Fotherby, K. A. Kelly, S. G. Priestman

## Abstract

The efficacy of high-dose intramuscular MPA therapy in controlling progressive measurable metastatic breast carcinoma was assessed in 32 women. In addition serial measurements of MPA blood levels were carried out in 20 of the patients and subjective effects of treatment were monitored in detail in 18 of the women. Overall 6 patients (19%) gained an objective response and a further 7 (22%) experienced disease stasis from 4-17 months whilst on treatment. Significant differences in serum MPA levels were seen between responders and non-responders, objective tumour shrinkage only being seen in those patients who rapidly attained, and sustained, blood levels in excess of 100 ng ml-1. Subjective assessment showed no evidence of a euphoriant effect of MPA therapy in the non-responders group.


*Br. J. Cancer* (1984), **50**, 363 -366

### An evaluation of high-dose medroxyprogesterone acetate (MPA) therapy in women with advanced breast cancer

J.R. Johnson[1], T.J. Priestman[1], K. Fotherby[2], K.A. Kelly[3] & S.G. Priestman[1]

[1]*Department of Radiotherapy and Oncology, Dudley Road Hospital, Birmingham, B18 7QH;* [2]*Department of Steroid Biochemistry, Royal Postgraduate Medical School, Hammersmith Hospital, London W12 OHS;* [3]*CRC Clinical Trials Unit, University of Birmingham, Birmingham B15 2TH, UK.*

**Summary** The efficacy of high-dose intramuscular MPA therapy in controlling progressive measurable metastatic breast carcinoma was assessed in 32 women. In addition serial measurements of MPA blood levels were carried out in 20 of the patients and subjective effects of treatment were monitored in detail in 18 of the women. Overall 6 patients (19%) gained an objective response and a further 7 (22%) experienced disease stasis from 4–17 months whilst on treatment. Significant differences in serum MPA levels were seen between responders and non-responders, objective tumour shrinkage only being seen in those patients who rapidly attained, and sustained, blood levels in excess of $100\,ng\,ml^{-1}$. Subjective assessment showed no evidence of a euphoriant effect of MPA therapy in the non-responders group.

Reports of high-dose Medroxyprogesterone acetate (MPA) administration in the treatment of advanced breast cancer began to appear in the mid 1970s and in 1979 a review of the published data concluded that an objective remission rate of 40% was seen in women who had not previously received cytotoxic therapy (Ganzina, 1979). In addition to objective benefit the relative lack of toxicity of MPA combined with its ability to relieve pain (Pannuti *et al.,* 1979), improve performance status (Ganzia, 1979), and increase appetite and body weight (Cavalli *et al.,* 1983) led to the view that treatment carried a significant subjective benefit. Clinical trials showed that daily intramuscular doses of MPA of 500 mg or more were necessary for a significant incidence of response (Ganzina, 1979), but pharmacokinetic studies have indicated considerable differences in bioavailability between patients (Salimtschik *et al.,* 1980). The present study set out across the objective response to high-dose MPA in women with advanced breast cancer and also to monitor both serum MPA levels and subjective effects of treatment in order to identify any correlations between these three parameters.

### Materials and methods

The study was designed as an open, single group assessment and women with evaluable, progressing, locally recurrent or metastatic carcinoma of the breast which had failed to respond to, or relapsed after, conventional therapy were enrolled.

Medroxyperogesterone acetate (Farlutal, Farmitalia Carlo Erba, 500 mg in 2.5 ml. suspension) was administered by deep i.m. injection into the gluteal region at a dose of 500 mg for 28 days. This induction phase was followed by maintenance therapy of 1000 mg i.m. once weekly. The majority of patients received treatment on an outpatient basis, injections being given by the District Nurse.

Pre-treatment assessment included a full physical examination, blood count, biochemical profile, chest X-ray and liver and bone isotope scans. Blood tests were repeated weekly during the induction phase and monthly thereafter. Other investigations were repeated when needed to assess response.

Objectives response was assessed at monthly intervals and UICC criteria were adopted (Hayward *et al.,* 1977). Complete response indicated disappearance of all known disease and partial response was a >50% decrease in measurable lesions and objective improvement in evaluable but non-measurable lesions, with no new lesions and no progressions of existing lesions. Those patients whose lesions decreased by <50% or increased by <25% were considered to have disease stasis. The UICC criteria do not specify a minimum duration in order for such regressions to be considered a response, but in the present study only those patients in whom remission or stasis was sustained for more than 3 months from the time of starting MPA were included in these categories.

Subjective response was monitored with a preliminary questionnaire prepared by the European Organisation for Research and Treatment of Cancer (EORTC) Study Group on Quality of Life. This was a verbal self-rating scale made up of 36 questions, 12 relating to purely

Correspondence: T.J. Priestman
Received 3 February 1984; accepted 12 June 1984.

© The Macmillan Press Ltd., 1984

physical parameters, two groups of 10 and 12 questions respectively examining positive and negative aspects of mood and the last two questions aimed at a global assessment of the patient's feelings. The development and validation of the questionnaire has been described elsewhere (Linssen *et al.*, 1982; Stewart & van Dam, 1983) but it has not previously been used to prospectively monitor the subjective effects of a specific treatment. Questionnaires were completed before treatment, at weekly intervals during the induction phase and monthly thereafter for a total of three months.

Medroxyprogesterone acetate levels were measured in the serum samples by radioimmunoassay (Shrimanker *et al.*, 1978), blood being taken before treatment, weekly during induction and monthly thereafter. Following venepuncture the blood was allowed to clot and the serum separated and stored at $-20°$ until analysed. The intra-assay variation was $<10\%$ and the inter-assay variation $<15\%$. The specificity of the assay has been previously reported as $>80\%$ (Mathrubutham & Fotherby, 1981) and in the present series most samples were completely specific with $\sim 30\%$ of the specimens showing small amounts (always $<20\%$) of other material reacting with the antiserum.

### Results

Thirty-two women entered the study, two died from rapidly progressive disease before completion of induction therapy, but have been included in the overall analysis. All patients had received extensive prior therapy (surgery in 24 cases, radiotherapy in 28, cytotoxic drugs in 23, tamoxifen in 31, other hormones in 15 and interferon in 3). The mean age of the group was 63 years (range 37–76) and all patients were post-menopausal.

Six patients (19%) had an objective response to treatment with 3 complete and 3 partial remissions. Details of the response rate for individual sites of disease are given in Table I. The mean duration of response was 12 months (range 8–16+), measured from the time of commencing MPA. One patient died at 14 months from other causes whilst still in remission and two others remain in remission at 9 and 16 months respectively. A further 7 women (22%) experienced disease stasis whilst receiving MPA. The average duration of stasis was 12 months (range 4–17).

**Table I** Response by site

| | *CR* | *PR* | *No change* | *Progression* |
|---|---|---|---|---|
| Soft tissue | | | | |
| breast ($n=13$) | — | — | 6 | 7 |
| cutaneous ($n=26$) | 4 | 2 | 9 | 11 |
| nodes ($n=20$) | 3 | 4 | 6 | 7 |
| Bone ($n=13$) | — | 1 | 3 | 9 |
| Lung ($n=9$) | — | 1 | — | 8 |
| Liver ($n=4$) | — | — | — | — |

Data on MPA plasma levels were available for 20 patients (5 of whom had objective response, 4 disease stasis and 11 progressive disease). In responders there was a rapid rise in blood levels to above 150 ng ml$^{-1}$ by the end of the induction period whereas the remaining patients had an average level of only 70 ng ml$^{-1}$ at this time (Figure 1). The difference in values between responders and the disease-stasis/progression group was statistically significant (Student *t* test) at all time points up to 12 weeks after commencing treatment (Table II). By this stage most of the non-responders had come off treatment, by 20 weeks all four of the disease-stasis group had reached blood levels in excess of 100 ng ml$^{-1}$.

**Figure 1** Title: Mean MPA blood level and response.

Scores for subjective assessment over the first 3 months of treatment were available for 5 patients who showed an objective response and 13 with progressive disease. A preliminary analysis showed a tendency for the overall scores for the responders to increase during treatment whereas those of the non-responders declined (Priestman, 1984). These global scores included a wide variety of factors and, in order to address the question of whether there was any euphoriant effect from the MPA therapy apparent in the non-responders, the nine parameters on the questionnaire specifically relating to the patient's feeling of well-being were analysed separately. (These were irritability, worry,

**Table II** MPA serum levels and response

| Time weeks | MPA serum level ng ml$^{-1}$ (mean & s.d.) responders (n=5) | non-responders & stasis (n=13) | Significance P |
|---|---|---|---|
| 0 | 0 | 0 | — |
| 1 | 45±30 | 17±12 (13)[a] | <0.05 |
| 2 | 76±32 | 40±23 (13) | <0.05 |
| 3 | 111±26 | 57±28 (13) | <0.05 |
| 4 | 153±21 | 69±30 (13) | <0.01 |
| 8 | 125±37 | 77±38 (11) | <0.05 |
| 12 | 135±43 | 88±27 (7) | NS |
| 16 | 158±49 | 107±23 (6) | NS |

[a]Figures in parenthesis give number of patients. The decline in numbers after 4 weeks is due to non-responders coming off treatment.

depression, anxiety, tension and four questions on general mood and feelings).

The results are shown in Figure 2 and, although scores for responders rise initially (indicating improved wellbeing) whilst the non-responders scores remain essentially static, there is no statistical difference between the two groups at any point in time (Wilcoxon rank sum test).

**Figure 2** Title: Scores for well-being during MPA treatment.

Twenty-two patients experienced side effects during treatment and in three these were of sufficient severity to stop treatment (Table III). The elevation of haemoglobin was greater than 1 mg dl$^{-1}$ in 15 patients and in 7 of these it was >2 mg dl$^{-1}$ but no symptoms resulted from this increase. The syndrome of trembling with or without muscle cramps and sweating usually only appeared after 2 to 3 months therapy and was mild in 6, moderate in 3 and severe in 3 cases. All but one of the patients with moderate or severe symptoms had plasma MPA levels greater than 130 ng ml$^{-1}$, and in all instances symptoms disappeared following reduction in MPA dose. Seven patients had an increase of more than 5% of their pre-treatment body weight, a further 11 had smaller weight gains. One patient developed central chest pain after 6 months of treatment and an ECG confirmed myocardial infarction, whilst a second had increased frequency of previously noted anginal attacks. One patient became myxoedematous during treatment but another, who was on treatment for hypothyroidism, found her thyroxine requirement to be halved. In one patient who developed a gluteal abscess at the injection site, this was sufficiently severe to necessitate admission to hospital for intensive supportive care.

**Table III** Side effects of high-dose MPA

| | | |
|---|---|---|
| Haemoglobin increase | 22 patients | (47%) |
| Trembling/muscle cramps/ sweating | 12 patients | (38%) |
| Weight gain | 7 patients | (22%) |
| Cushingoid facies | 4 patients | (13%) |
| Gluteal abscess | 1 patient | (3%) |

### Discussion

Previous studies of high-dose MPA in advanced breast cancer have reported response rates ranging from 28% (Mattson, 1978) to 46% (Pannuti *et al.*, 1979) in patients who had not previously received cytotoxic therapy and this figure has risen to over 70% when patients were selected according to oestrogen-receptor status (Mattson, 1980). In comparison with these figures the results in the present series are, at first sight, somewhat disappointing, but this lower response rate is probably due to the heavy pre-treatment of the patients reflecting a more advanced stage of disease. Previous evaluations of high-dose MPA have commented on the beneficial effects on bone metastases with objective response rates in excess of 50% being reported in patients with skeletal secondaries (Pannuti *et al.*, 1979; Robustelli della Cuna *et al.*, 1978) whereas in this series only 1/13 patients with bone involvement gained objective benefit. All previous series have commented on the higher response rate seen in soft tissue recurrence as compared with visceral secondaries and the present results reinforce this observation. When responses did occur, however, they were of worthwhile duration and the induction of disease stasis in 21% of patients for an average of 12 months should probably also be considered a therapeutic advantage.

There was no relationship between MPA serum levels and race, body weight or liver function. Within one week of commencing therapy the responders had reached significantly higher serum levels than the remaining patients and their values remained significantly higher up to 16 weeks. Interpretation of the levels at and beyond this time is obscured by dose reductions, due to toxicity, and the fall off in the numbers of non-responders due to patients coming off treatment because of progressive disease. The shape of the curves for the two patient groups suggests that the responders may have been slower metabolisers of MPA, thus rapidly achieving high blood levels, and the possibility of there being different patterns of MPA metabolism which might be of therapeutic significance merits further investigation.

A universally accepted and completely validated system for monitoring subjective response has still to be defined and it would probably be premature to consider the results of any such analysis as conclusive. Given this caveat it was still considered important to try to identify whether MPA therapy was associated with a euphoriant effect. For this reason only these aspects of the EORTC questionnaire relating specifically to mood and well-being have been considered in the present analysis, excluding those parameters relating to physical status, symptoms of disease and side-effects of therapy. On the basis of the data presented here no improvement in well-being could be identified in the non-responders and only a minimal transient benefit was recorded among those who showed objective regression of disease.

All the side effects seen in the present study have been reported previously. The significance of cardiovascular complications (myocardial infarction and increased frequency of anginal attacks) in two patients, and changes in thyroid function in two further women, remains to be determined with relation to MPA therapy. Compliance was not a problem and treatment was generally carried out on an outpatient basis, injections being given by the District Nurse. Although one patient did develop a severe gluteal abscess, this was only one case in a series involving over 1500 injections.

These results indicate that MPA has useful activity in either arresting disease progression or bringing about objective remission of worthwhile duration in women with end-stage breast carcinoma.

We would like to thank Ms Pamela Perry of Farmitalia Carlo Erba for her help in establishing this study and for providing the Farlutal.

**References**

CAVALLI, F., GOLDHIRSCH, A., JUNGI, F., MARTZ, G. & ALBERTO, P. (1983). Low versus high dose medroxyprogesterone acetate in the treatment of advanced breast cancer. In: *Role of Medroxyprogesterone Acetate in Endocrine Related Tumours.* (Eds. Campio, Cuna & Taylor). New York: Raven, Vol. 2, p. 69.

GANZINA, F. (1979). High-dose medroxyprogesterone acetate (MPA) treatment in advanced breast cancer. A review. *Tumori*, **65**, 563.

HAYWARD, J.L., CARBONE, P.P., HEUSON, J.C., KUMAOKA, S., SEGALOFF, A. & RUBENS, R.D. (1977). Assessment of response to therapy in advanced breast cancer. *Eur. J. Cancer*, **13**, 89.

LINSSEN, A.C.G., HANEWALD, G.P., HUISMAN, S. & VAN DAM F.S. (1982). The development of a well-being (quality of life) questionnaire at the Netherlands Cancer Institute. In: *Proceedings from the 3rd Workshop EORTC Study Group on Quality of Life.* (Ed. Beckmann), Denmark: Odense University, p. 82.

MATHRUBUTHAM, M. & FOTHERBY, K. (1981). Medroxyprogesterone acetate in human serum. *J. Steroid Biochem.*, **14**, 783.

MATTSON, W. (1978). High-dose medroxyprogesterone acetate treatment in advanced mammary carcinoma. *Acta Radiol. Oncol.*, **17**, 387.

MATTSON, W. (1980). A phase III trial of treatment with tamoxifen versus treatment with high-dose medroxyprogesterone acetate in advanced post-menopausal breast cancer. In: *Role of Medroxyprogesterone in Endocrine-Related Tumours.* (Eds. Iacobelli & DiMarco), New York: Raven, p. 65.

PANNUTI, F., MARTONI, A., DI MARCO, A.R. & 11 others. (1979). Prospective randomised clinical trial of two different high dosages of medroxyprogesterone acetate (MPA) in the treatment of metastatic breast cancer. *Eur. J. Cancer*, **15**, 593.

PRIESTMAN, T.J. (1984). Quality of life after cancer chemotherapy. *J. Roy. Soc. Med.*, (In press).

ROBUSTELLI DELLA CUNA G., CALCIATA, A., STRADA, B.M., BUMMA, C. & CAMPIO, L. (1978). High dose medroxyprogesterone acetate (MPA) treatment in metastatic carcinoma of the breast. *Tumori*, **64**, 143.

SALIMTSCHIK, M., MOURIDSEN, H.T., LOEBER, J. & JOHANSSON, E. (1980). Comparative pharmacokinetics of medroxyprogesterone acetate administered by oral and intramuscular routes. *Cancer Chemother. Pharmacol.*, **4**, 267.

SHRIMANKER, K., SAXENA, B.N. & FOTHERBY, K. (1978). A radioimmunoassay for serum medroxyprogesterone acetate. *J. Steroid Biochem.*, **9**, 359.

STEWART, A.L. & VAN DAM F.S. (1983). Quality of life outcomes selected for study in clinical trials of lung cancer, oesophageal cancer and breast cancer. In: *Proceedings from the 4th Workshop EORTC Study Group on Quality of Life.* (Ed. Beckmann), Denmark: Odense University, p. 72.